# Dupilumab rapidly improves asthma control in predominantly anti‐IL5/IL5R pretreated Austrian real‐life severe asthmatics

**DOI:** 10.1002/iid3.434

**Published:** 2021-05-07

**Authors:** Andreas Renner, Katharina Marth, Karin Patocka, Marco Idzko, Wolfgang Pohl

**Affiliations:** ^1^ Karl Landsteiner Institute for Clinical and Experimental Pneumology Hietzing Hospital Vienna Austria; ^2^ Department of Pulmonology Medical University of Vienna Vienna Austria; ^3^ Present address: Katharina Marth, Individualized Drug Therapy Research Program, Faculty of Medicine, Helsinki University Hospital University of Helsinki Helsinki Finland

## Abstract

Dupilumab is a monoclonal antibody against the IL‐4 receptor alpha which has shown efficacy in T2 high severe asthmatics in phase 3 randomized controlled trials. The purpose of this real‐life study is to demonstrate the real‐life effectiveness of dupilumab in Austrian severe asthma patients. We retrospectively analyzed all patients receiving dupilumab at our severe asthma clinic. Thirteen patients have so far received dupilumab at our center. The primary outcome, asthma control questionnaire 6‐item scale at 2 weeks, improved by 0.57 points (*p* = .014), which is statistically and clinically significant. Similarly, the asthma control test at 4 weeks improved by 3.91 points (*p* = .024), also statistically and clinically significant. Improvements in forced expiratory volume in 1 s at 2 weeks were neither statistically, nor clinically significant. Improvements at 4 weeks (+220 ml, *p* = .041), and 3 months (+229 ml, *p* = .006), were statistically significant and clinically borderline significant. No severe adverse events or hypereosinophilia were observed. No adverse events led to treatment discontinuation. Most patients (85%) had previously received monoclonal antibody treatment for severe asthma. Previous monoclonal antibody treatment had been discontinued in these patients due to a lack of clinical response. Dupilumab is effective and safe in Austrian real‐life severe asthmatics. It provides a possible treatment strategy for T2 high severe asthmatics who do not qualify for anti‐immunoglobulin E or anti‐IL5/IL5R monoclonal antibody treatments or do not adequately respond to these.

## Graphical abstract

This is a real‐life study that shows that dupilumab is effective and safe in Austrian real‐life severe asthmatics. It rapidly improved asthma control. These improvements are comparable to previous randomized controlled trials.

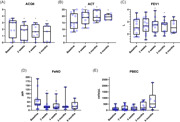

1

Dupilumab is a monoclonal antibody (mAb) against the IL‐4 receptor alpha (IL‐4Rα). Randomized controlled trials (RCTs) have shown the efficacy of dupilumab in severe, type 2 high, asthma.^1^ So far, only one real‐life study on dupilumab in severe asthma has been published.^2^ Dupin et al.^2^ showed clinically meaningful and statistically significant improvements in lung function (forced expiratory volume in 1 s, FEV1) and asthma control (asthma control test, ACT).

The purpose of this retrospective, real‐life study is to add evidence on the real‐life effectiveness of dupilumab in severe asthma. This is, to our knowledge, the second worldwide, and first Austrian real‐life study on dupilumab in severe asthma.

All patients who received at least one application of dupilumab at our tertiary care severe asthma clinic were included for analysis (see methods in the Online Supporting Information). Approval from the local ethics committee was granted. Due to the retrospective study design, written informed consent was not needed.

Thirteen patients have so far received dupilumab at our center. The baseline characteristics are summarized in Table 1. Three of these patients (23%) received maintenance oral corticosteroids (OCS) at treatment initiation. The primary outcome, ACQ6 at 2 weeks, improved by 0.57 points (*n* = 10; 2.50 ± 1.41 at baseline and 1.53 ± 1.22 at week 2, *p* = .014, see Figure 1A). Considering the minimally clinically important difference (MCID) of 0.5 in the ACQ6,^3^ this improvement is not only statistically but also clinically significant. Similarly, ACT at 4 weeks improved by 3.91 points (*n* = 11; 14.82 ± 5.69 at baseline and 18.73 ± 4.96 at week 4, *p* = .024, see Figure 1B), exceeding the MCID of 3 points.^4^ This statistically and clinically significant improvement was sustained at 3 months for both ACQ6 (*n* = 7; −0.82; 2.20 ± 1.38 at baseline and 1.38 ± 1.29 at 3 months, *p* = .045) and ACT (*n* = 8; + 4.00; 16.00 ± 5.98 at baseline and 20.00 ± 4.14 at 3 months, *p* = .041). ACT was still significantly and clinically significantly improved at 6 months (*n* = 6; + 6.83; 12.50 ± 4.68 at baseline and 19.33 ± 2.07 at 6 months, *p* = .044), due to missing data points formal analysis for ACQ6 was not performed for this timepoint.

**Figure 1:**
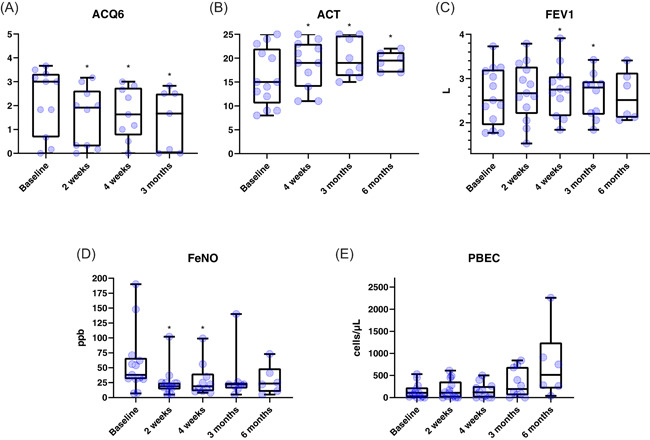
Box and whiskers plots of ACQ6 (A), ACT (B), FEV1 (L) (C), FeNO (ppb, D), and PBEC (E) at baseline and subsequent visits. Boxes show median and interquartile range, whiskers maximum and minimum. ACQ6, asthma control questionnaire 6‐item scale; ACT, asthma control test; FEV1, forced expiratory volume in 1 s; FeNO, fraction of exhaled nitric oxide; PBEC, peripheral blood eosinophilic count. *p < .05

**Table 1:** Patients' characteristics at baseline

Patients with severe asthma who received at least one treatment with dupilumab (*N* = 13)	
Age (y), (±*SD*)	47 (±12)
Age at diagnosis (y), (±*SD*)	51 (±19)
Former smokers, all, *N* (%)	4 (31)
Female patients, *N* (%)	4 (31)
Body mass index (kg/m^2^), (±*SD*)	27.6 (±4.6)
CRSwNP, *N* (%)	8 (62)
Clinically relevant allergies, *N* (%)	11 (85)
Annual exacerbation rate, median (IQR)	2 (2–4)
Patients on maintenance OCS, *N* (%)	3 (23)
OCS dose in patients on maintenance OCS, mg in prednisolone equivalent, (±*SD*)	15.2 (±8.8)
Any asthma‐mAB treatment before, *N* (%)	11 (85)
Omalizumab before, *N* (%)	4 (41)
Reslizumab before, *N* (%)	4 (41)
Mepolizumab before, *N* (%)	3 (23)
Benralizumab before, *N* (%)	7 (54)
FEV_1_ (L) pre BD, (±*SD*)	2.61 (±.65)
FEV_1_(% predicted), (±*SD*)	69 (±21)
ACT, (±*SD*)	16 (±5.9)
Patients with an ACT ⩽ 19, *N* (%)	8 (62)
ACQ6, (±*SD*)	2.20 (±1.38)
PBEC (cells/µl), (IQR)	110 (10–160)
FeNO (ppb), (IQR)	38 (32–59)

Abbreviations: ACQ6, asthma control questionnaire 6‐item scale; ACT, asthma control test; BD, bronchodilator; CRSwNP, chronic rhinosinusitis with nasal polyps; FeNO, fraction of exhaled nitric oxide; FEV_1_, forced expiratory volume in 1 s; IQR, interquartile range; mAB, monoclonal antibody; OCS, oral corticosteroids; PBEC, peripheral blood eosinophil count; *SD*, standard deviation.

FEV1 improved by 97 ml at 2 weeks (*n* = 13; 2613 ± 649 ml at baseline and 2710 ± 651 ml at week 2, *p* = .176, see Figure 1C), which was neither statistically nor clinically significant, considering an MCID of 230 ml.^5^ FEV1 improvements at 4 weeks (*n* = 11; + 220 ml; 2517 ± 661 ml at baseline and 2737 ± 598 ml at week 4, *p* = .041), 3 months (*n* = 10; +229 ml; 2396 ± 553 ml at baseline and 2625 ± 505 ml at 3 months, *p* = .006) were statistically significant and clinically borderline significant. At 6 months FEV1 improvement was only minimal (*n* = 6; +34 ml; 2578 ± 601 ml at baseline and 2612 ± 563 ml at 6 months, *p* = .889).

All three patients who received maintenance OCS at baseline were able to reduce their OCS dose by at least 50% at 3 months while improving asthma control. Eight patients had chronic rhinosinusitis with nasal polyps (CRSwNP) at baseline. Of those patients, six (75%) showed clinically relevant CRSwNP improvements after 1 month. These improvements continued until the end of follow‐up in five of those patients. In one patient, symptoms returned to baseline levels at 3 months. Two patients had no relevant change in CRSwNP symptoms under dupilumab treatment. During dupilumab treatment, the annualized exacerbation rate was significantly reduced (0 [0–1], *p* = .01) compared to the pretreatment annual exacerbation rate (2 [2–4]). No severe adverse events were observed. No adverse events were linked to the injection and/or hypereosinophilia. No adverse events led to treatment discontinuation. See results on biomarkers in the Online Supporting Information.

Dupilumab rapidly improved asthma control in real‐life severe asthmatics. This improvement was both statistically and clinically significant. The clinical improvements seen here are comparable to both RCTs^1^ and the previously published real‐life study by Dupin et al.,^2^ with slightly less pronounced improvements in FEV1. To our knowledge, this is the first real‐life study demonstrating meaningful improvements in asthma control within 2 weeks of treatment initiation. FEV1 improvements, while clinically and statistically significant at 4 weeks and 3 months, were not seen at 6 months. In the RCT by Castro et al.^1^ FEV1 improvements persisted until Week 52. As asthma control improvements (both ACQ6 and ACT) did not deteriorate at 6 months in our study a loss of effectiveness is unlikely. Further real‐life studies with a bigger sample size as well as a longer follow‐up are needed to clarify this discrepancy. It is especially noteworthy that most of our patients (85%) had previously received mAB treatment for severe asthma, most of them benralizumab (51%). Previous mAB treatment had been discontinued in these patients due to a lack of clinical response. These findings are comparable to real‐life data by Mümmler et al.^6^ Similar to previous studies,^1^, ^2^ hypereosinophilia was seen in some patients but did not affect treatment response, nor manifest in adverse events. The main limitations of this study are the small sample size, as well as the retrospective study design. Due to consecutive treatment initiation at our clinic as well as a fixed end date for data collection the sample size is even smaller at 6 months (*n* = 6). For that reason, the evidence on exacerbation rate improvement is weak and needs to be confirmed in real‐life studies with a longer follow‐up period.

Dupilumab is effective and safe in Austrian real‐life severe asthmatics. It provides a possible treatment strategy for T2 high severe asthmatics who do not qualify for anti‐immunoglobulin E or anti‐IL5/IL5R mABs or do not adequately respond to these.

## CONFLICT OF INTERESTS

Prof. Pohl reports personal fees from Astra Zeneca, Boehringer Ingelheim, Chiesi, GSK, GILEAD, MedImmune, Menarini, Novartis, Sciotec, and Teva, outside the submitted work.

## AUTHOR CONTRIBUTIONS

*Conception and design of the study, data analysis, interpretation of the data, wrote the first draft of the manuscript*: Andreas Renner. *Critically reviewed and edited the manuscript, data acquisition*: Katharina Marth and Karin Patocka. *Critically reviewed and edited the manuscript*: Marco Idzko. *Conception and design of the study, data acquisition, interpretation of the data, critically reviewed and edited the manuscript*: Wolfgang Pohl.

## Supporting information

Supporting information.

## Data Availability

The data that support the findings of this study are available on reasonable request from the corresponding author. The data are not publicly available due to privacy or ethical restrictions.
