# Supplementary material for: Dupilumab rapidly improves asthma control in predominantly anti‐IL5/IL5R pretreated Austrian real‐life severe asthmatics
Source: Immun Inflamm Dis. 2021 May 7;9(3):624–7. doi: 10.1002/iid3.434 (PMC8342226; doi:10.1002/iid3.434)
Supplement: Supplementary file 1 — Supporting information. [file IID3-9-624-s001.docx]

**Dupilumab rapidly improves asthma control in predominantly anti-IL5/IL5R pre-treated Austrian real-life severe asthmatics. - Online supplementary**

Methods:

Patient-reported asthma control measures (ACQ6, ACT), FEV1, peripheral blood eosinophilia count (PBEC), fraction of exhaled nitric oxide (FeNO), oral corticosteroid dose (OCS, if applicable), exacerbations, as well as adverse events were collected at baseline and every subsequent visit. The primary outcome was pre-defined as an improvement in ACQ6 after 2 weeks of treatment initiation. We believe patient reported outcomes (ACQ6 and ACT) best reflect improvement in clinical practice. Furthermore, ACQ6 can measure improvement in asthma control within a week. The focus of this study was pre-defined to evaluate rapid clinical improvement. For these reasons we deemed ACQ6 after 2 weeks to be the most appropriate primary outcome in this study.

Comparisons were made using a paired 2-tailed t-test or Wilcoxon signed-rank test, depending on normality. Normality was assessed using the Shapiro-Wilk test. All statistical analyses were performed using SPSS Version 25.0 (IBM Corporation, Armonk, NY).

Results:

Due to the small number of patients receiving OCS at baseline (n=3), no formal analysis of a potentially steroid sparing effect was conducted.

Due to the low number of patients who have so far received dupilumab longer than 6 months (n=5), no formal analysis was performed for further timepoints. It is important to point out that no patient has so far discontinued dupilumab. Treatment and data collection are being continued and analysis of long-term outcomes will be performed.

Changes in PBEC were neither clinically relevant nor statistically significant. There was a noticeable but not statistically significant increase in PBEC (cells/µL) at 6 months (n=6; +528; 222 [±230] at baseline and 750 [±809] at 6 months, p=0.200). Three patients developed hypereosinophilia exceeding 1000 cells/µL, including one patient on OCS (see Fig. 1E). Hypereosinophilia did not impair asthma control, nor result in adverse events. FeNO decreased by 33 ppb (n=13; 59 ppb [±53] at baseline and 25 ppb [±25] at 2 weeks, p=0.004, see Fig 1D), which is statistically significant. This decrease was sustained until 6 months, although not statistically significant at 3 and 6 months, possibly due to smaller sample size (n=8 and n=6, data not shown).
